# Remote cortical degeneration related to structural connectivity following recent small subcortical infarcts

**DOI:** 10.1016/j.nicl.2026.103987

**Published:** 2026-03-20

**Authors:** Youjie Wang, Jingyu Cui, Yuying Yan, Tang Yang, Yue Yuan, Rumei Lei, Rongfeng Luo, Bo Wu, Shuai Jiang

**Affiliations:** Department of Neurology, West China Hospital of Sichuan University, No. 37 Guo Xue Xiang, Chengdu 610041, China

**Keywords:** Secondary degeneration, Recent small subcortical infarcts, Diffusion tensor imaging

## Abstract

**Background:**

Secondary cortical degeneration caused by the remote effects of subcortical infarcts has been implicated in long-term outcomes after acute ischemic stroke. However, this process remains insufficiently studied in recent small subcortical infarcts (RSSI). We aimed to verify RSSI-induced cortical damage, determine whether it can be captured by neuroimaging markers, and explore its association with clinical outcomes.

**Methods:**

RSSI patients with longitudinal Magnetic Resonance Imaging (MRI) were included. Cortical degeneration was assessed using linear mixed-effects models, incorporating a direct approach based on individual diffusion weighted imaging and an indirect approach using the normative connectome from the Human Connectome Project (HCP). Principal component analysis (PCA) was employed to extract features of cortical alterations. The resulting component scores were used in general linear models to assess associations with neuroimaging markers and clinical outcomes.

**Results:**

A total of 76 RSSI patients were analyzed. RSSI was found to induce progressive cortical thinning and volume loss in structurally connected regions. PCA identified a component reflecting parenchymal atrophy associated with diffusion-based markers of white matter integrity, as well as the presence of track/cap signs. Moreover, faster cortical degeneration in lesion-connected regions was significantly associated with a greater increase in Hamilton Anxiety Rating Scale (HAMA) scores (β = -2.38, 95% CI = -4.30 – -0.47, *p* = 0.017).

**Conclusions:**

RSSI induces secondary cortical damage through structurally connected fiber tracts, which is detectable by neuroimaging markers of white matter integrity. These regional cortical alterations may be relevant to post-stroke outcomes and require validation in larger longitudinal studies.

## Highlights

• RSSI causes remote cortical atrophy via structural connectivity.
• White matter integrity predicts cortical atrophy rate.
• Atrophy in lesion-connected cortex relates to HAMA worsening.
• Subject-specific dMRI and normative connectomes yield similar findings.

## 1 Introduction

Recent small subcortical infarcts (RSSI), also referred to as acute lacunar infarcts, represent an acute manifestation of cerebral small vessel disease (cSVD), which accounts for approximately 25% of ischemic strokes (Duering et al., 2023). RSSI is typically identified on diffusion-weighted imaging (DWI) as a recent infarct accompanied by corresponding neurological deficits. Although small in size, RSSI is associated with all-cause mortality rates comparable to other ischemic stroke types and with high rates of recurrent infarction, cognitive impairment, and functional decline (Cheng et al., 2025, Portegijs et al., 2022). The mechanisms and risk factors underlying these unfavorable outcomes remain inadequately understood.

Secondary degeneration from the remote effects of subcortical lesions is considered a pivotal mechanism influencing post-stroke outcomes (Carrera and Tononi, 2014). Studies have examined dynamic cortical changes following subcortical stroke, with some focusing on identifying cortical regions exhibiting structural alterations and their anatomical relationships with the lesion site (Cai et al., 2016). Advances in diffusion tensor imaging (DTI) enables mapping of patient-specific structural connectomes to assess degeneration in cortical regions anatomically connected to the infarct lesion (Duering et al., 2012), as well as broader disruptions in brain networks. Nevertheless, these studies were conducted in relatively small cohorts and included patients with heterogeneous etiologies of subcortical infarction (Duering et al., 2015). Similar approaches have also been implemented using normative connectomes derived from healthy individuals in the Human Connectome Project (HCP) (Kuceyeski et al., 2014). More recently, these methods have been applied to investigate remote damage associated with other subcortical pathologies, such as white matter hyperintensities (WMH) (Li et al., 2023, Zhang et al., 2024). However, few studies have focused on RSSI, a stroke subtype with relatively homogeneous etiology, with over 80% of cases attributed to small vessel pathology (Duering et al., 2023). Meanwhile, most existing work has relied exclusively on either patient-derived or normative connectomes, rather than integrating and comparing both.

Certain RSSI lesion evolution characteristics, such as cavitation and hemorrhagic foci, have been linked to outcomes (Jiang et al., 2025). A recently proposed imaging marker on fluid attenuated inversion recovery (FLAIR) image, the track/cap sign, has also been associated with cSVD progression and functional outcomes (Cheng et al., 2025). Beyond these visually apparent features, diffusion-based indices, such as fractional anisotropy (FA) and mean diffusivity (MD), are increasingly recognized as sensitive indicators of cSVD burden and disease progression (Benjamin et al., 2016, Egle et al., 2022). However, their relationship with secondary cortical damage in RSSI remains unexplored.

Building upon these observations, we integrated longitudinal structural magnetic resonance imaging (MRI) with single-shell diffusion data to investigate: (1) whether RSSI leads to remote cortical alterations, using both direct (patient-specific DWI) and indirect (HCP-based normative connectome) approaches, as both strategies characterize lesion-related structural connectivity; (2) which factors, including cSVD burden, lesion evolution, and white matter injury, are associated with the extent of remote cortical damage; and (3) whether such cortical damage correlates with clinical outcomes.

## 2 Methods

### 2.1 Participants

We retrospectively reviewed data from a prospective RSSI cohort of the Department of Neurology at West China Hospital between July 2018 and February 2025 (Jiang et al., 2022, Jiang et al., 2020). Patients were included if they (1) had a first-ever RSSI in the penetrating arterial territory, confirmed on baseline DWI with corresponding neurological deficits; and (2) had available multi-modality MRI data, including 3D T1-weighted and axial T2 FLAIR images at both baseline and follow-up (performed approximately one year after the infarction), as well as follow-up single-shell DWI.

Exclusion criteria were as follows: (1) evidence of cardioembolism (e.g., atrial fibrillation or valvular heart disease); (2) ≥ 50% stenosis of the ipsilateral intracranial internal carotid artery or relevant extracranial arteries on computed tomography angiography (CTA) or magnetic resonance angiography (MRA); (3) presence of other non-atherosclerotic vasculopathies (e.g., vasculitis or moyamoya disease); or (4) poor MRI quality or processing errors during image analysis.

Demographic characteristics and vascular risk factors were collected for all participants at baseline. Functional outcomes were assessed using the modified Rankin Scale (mRS). Global cognitive performance was measured with the Montreal Cognitive Assessment-Beijing version (MoCA), and executive function was evaluated using completion times on the Shape Trail Test Part A (STT-A) and Part B (STT-B). Depression and anxiety severity were assessed using the 17-item Hamilton Depression Rating Scale (HAMD) and the 14-item Hamilton Anxiety Rating Scale (HAMA), respectively. These assessments were performed at both baseline and follow-ups on the same day that the MRI scans were acquired.

The study was approved by the Medical Ethics Committee of West China Hospital, Sichuan University (No.2018521), and conducted in accordance with the ethical principles expressed in the Declaration of Helsinki. Written informed consent was obtained from all participants prior to their enrollment in the study.

### 2.2 MRI protocol

All MRI scans were performed on a 3.0 T MR scanner (MAGNETOM Trio, Siemens, Erlangen, Germany) using a 32-channel head coil. Both baseline and follow-up scans were acquired on this identical platform to ensure consistency across time points. The 3D-T1-weighted image was acquired using a magnetization-prepared rapid gradient-echo (MPRAGE) sequence with the following parameters: echo time (TE) = 2.26 ms, repetition time (TR) = 1900 ms, inversion time = 900 ms, field of view (FOV) = 256 mm × 256 mm, matrix = 256 × 256, flip angle = 9°, and isotropic voxel size = 1 mm^3^. The T2 FLAIR image was collected with TE = 93 ms, TR = 9000 ms, inversion time = 2500 ms, flip angle = 130°, FOV = 196 mm × 220 mm, matrix = 456 × 512, and slice thickness = 6.5 mm. The single-shell DWI was acquired using a 2D echo-planar imaging (EPI) sequence with 20 diffusion gradient directions (*b* = 1000 s/mm^2^) and one volume without diffusion weighting (*b* = 0 s/mm^2^). Imaging parameters were: TE = 93 ms, TR = 6800 ms, flip angle = 90°, FOV = 230 mm × 230 mm, matrix size = 128 × 128, voxel size = 1.8 × 1.8 × 3.0 mm^3^, and number of averages = 2, resulting in a total of 42 volumes. Details of other MRI sequences used in this study have been described previously (Jiang et al., 2025).

### 2.3 cSVD score and vascular risk factor (VRF) score

cSVD imaging markers, including WMH, lacunes, cerebral microbleeds (CMB), and enlarged perivascular spaces (EPVS), were rated following the standards for reporting vascular changes on neuroimaging (STRIVE) criteria (Duering et al., 2023, Wardlaw et al., 2013). A total cSVD score ranging from 0 to 4 was calculated depending on these cSVD imaging markers: (1) presence of lacunes; (2) periventricular WMH Fazekas score of 3 or deep WMH Fazekas score of 2 or 3; (3) 2–4 grades EPVS in the basal ganglia; and (4) presence of CMB (Staals et al., 2014). A compound vascular risk factor (VRF) score ranging from 0 to 4 was derived by summing the presence of four common risk factors (hypertension, hyperlipidemia, diabetes, and current smoking) as documented in baseline medical records and patient self-report, with a score of 4 indicating the highest vascular risk burden (Cai et al., 2022).

### 2.4 Manual lesion segmentation

For all participants, RSSI lesions were manually delineated as binary masks on baseline *b* = 1000 DWI using MRIcroGL Software by an experienced neuroimaging researcher (YJ.W). Each lesion mask was subsequently linearly registered to the participant’s corresponding structural and diffusion spaces, and also nonlinearly normalized to the standard Montreal Neurological Institute (MNI) space for use in downstream analyses.

### 2.5 Neuroimaging data processing

Structural MRI data (T1-weighted images) were processed using FreeSurfer (version 7-dev) ‘recon-all’ pipeline with the longitudinal stream to generate an unbiased within-subject template and improve the stability of cortical surface reconstruction across time points (Reuter et al., 2010). From this process, we extracted unsmoothed surface-based measures, including cortical thickness, grey matter volume, surface area, mean curvature, and sulcal depth, hereafter referred to as thickness, volume, area, curv, and sulc for brevity, respectively. Cortical thickness reflects the distance between the white and pial surfaces and is sensitive to microstructural changes in cortical tissue. Grey matter volume represents the overall amount of cortical tissue. Surface area reflects the areal extent of the cortical sheet. Mean curvature and sulcal depth characterize cortical folding geometry, capturing morphological features of gyri and sulci (Fischl, 2012). These features were selected to serve as the primary metrics of interest in the present study due to tir widespread application in investigating disease-related cortical changes (Alhusaini et al., 2012, Demirci and Holland, 2022, Ronan et al., 2011), as well as their established utility in structural covariance network analyses as markers of cortical architecture (Sebenius et al., 2023). Additionally, T1-weighted images acquired at the follow-up time point for each participant underwent anatomical preprocessing using the ‘fsl_anat’ pipeline implemented in FMRIB Software Library (FSL), in order to obtain the nonlinear warp field for transformation to the standard MNI space.

For diffusion MRI preprocessing, denoising and Gibbs ringing artifact removal were first performed using MRtrix3 (Tournier et al., 2019). To correct for EPI distortions, we applied Synthesized b0 for Diffusion Distortion Correction (Synb0-DISCO), a deep-learning-based framework that synthesizes an undistorted *b* = 0 image from each participant’s T1-weighted image (Schilling et al., 2019). This synthetic image, together with the native distorted *b* = 0 image from the DWI data, was then processed using FSL’s TOPUP to estimate the susceptibility-induced off-resonance field. Finally, the fieldmap estimated by TOPUP was used in FSL’s eddy to perform motion and eddy current correction, yielding the preprocessed dataset.

The details of the derivation of quantitative neuroimaging markers are provided in the Supplementary Materials. Briefly, baseline total WMH volume was used as an indicator of cSVD burden (Wang et al., 2025). Lesion evolution characteristics, including cavitation and the presence of track/cap signs, were evaluated according to criteria previously described (Cheng et al., 2025, Jiang et al., 2025). DTI metrics, including FA and MD, were calculated in both normal-appearing white matter (NAWM) and lesion-connected tracts to serve as markers of white matter integrity. Additionally, to isolate the influence of lesion-related disconnection, FA and MD were calculated in non-connected normal-appearing white matter (ncNAWM), defined as NAWM excluding lesion-connected tracts. The peak width of skeletonized mean diffusivity (PSMD), designed to capture diffuse white matter damage, was used as an indicator of cSVD severity according to previous studies (Low et al., 2020).

### 2.6 Estimation of lesion-connected cortical region

#### 2.6.1 Direct estimation

The direct definition of lesion-connected surface region referenced the previous research, which has been validated in subcortical infarcts and white matter hyperintensities-connected area (Duering et al., 2012, Duering et al., 2015, Li et al., 2023, Zhang et al., 2024). Probabilistic tractography was performed with FSL, generating connectivity distributions from the infarction as seed voxels. Each voxel was assigned a value representing its connection strength to the lesion, yielding a tract density image. The image was then projected onto the individual’s native cortical surface space. To define surface regions with different levels of structural connectivity to the lesion, values at each vertex were normalized by the total number of generated streamlines (i.e., number of seed voxels × number of samples per voxel). A lower threshold of 3.8 × 10^−5^ was used to define the minimum connectivity level (low level). Among the remaining vertices, the top 50% and top 25% were classified as medium and high connectivity levels, respectively. All other cortical regions not included in the low-level region of interest (ROI) mask were defined as unconnected regions. To control for potential region-specific differences in cortical atrophy rates, a mirrored analysis was performed using the contralateral lesion region as the seed mask.

#### 2.6.2 Indirect estimation

The indirect estimation utilizes the normal tractography data from the HCP dataset to infer the potential impact of the lesion on the brain connectome and was originally introduced by Kuceyeski et al. (Kuceyeski et al., 2014). We applied the Network Modification (NeMo) toolkit (https://github.com/kjamison/nemo) to compute whole-brain Change in Connectivity (ChaCo) scores in MNI space. Briefly, a tractography database of 420 healthy individuals was generated using the probabilistic iFOD2 algorithm combined with anatomically constrained tractography (ACT). The voxel-wise ChaCo map was computed as the proportion of lesion-intersecting streamlines terminating in each voxel among all streamlines terminating in that voxel, and then averaged across subjects. The ChaCo maps were subsequently projected to the ‘fsaverage’ cortical surface using a precomputed transformation matrix implemented in the neuromaps toolbox (https://github.com/netneurolab/neuromaps). Additional information concerning the estimation of the RSSI-connected cortical region is presented in the Supplementary Material.

#### 2.6.3 Comparison between direct and indirect approaches

To evaluate the consistency between the direct and indirect approaches in delineating lesion-connected cortical regions, we examined the spatial correspondence of their connectivity maps. The normalized tract density image derived from the direct approach and the ChaCo map obtained from the indirect approach were both represented in surface space and averaged across subjects. Due to the positively skewed distribution of connectivity values, log transformation was applied prior to statistical comparison. Spatial correlations between the two approaches were assessed at both the vertex level and the regional level using the Desikan-Killiany atlas (34 regions per hemisphere). Spearman’s rank correlation coefficients were evaluated, and statistical significance was evaluated using spatial null models with 5000 permutations (Alexander-Bloch et al., 2018).

**Fig. 1:**
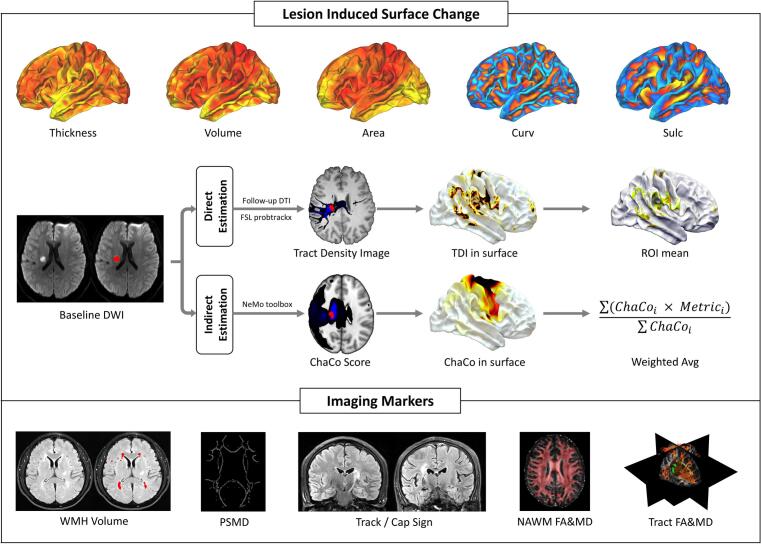
Schematic overview of the definition of RSSI-connected cortical regions and the neuroimaging markers used in the present study. Upper panel: Five cortical surface metrics were used to characterize cortical grey matter, including cortical thickness (thickness), gray matter volume (volume), surface area (area), mean curvature (curv), and sulcal depth (sulc). Lesion-connected cortical regions were identified using both direct and indirect estimation methods. In the direct approach, three levels of lesion-connected ROIs were derived from each patient's single-shell DWI data (red = high connectivity, green = medium connectivity, yellow = low connectivity), and average values of cortical metrics were extracted within each ROI. In the indirect approach, a normative connectome was used to compute the change in connectivity (ChaCo) value for each cortical vertex, which was then used to compute weighted averages of the cortical metrics. Lower panel: Summary of the neuroimaging markers analyzed in the study. TDI, tract density image; WMH, white matter hyperintensity; PSMD, peak width of skeletonized mean diffusivity; NAWM, normal-appearing white matter; FA, fractional anisotropy; MD, mean diffusivity. (For interpretation of the references to colour in this figure legend, the reader is referred to the web version of this article.)

Fig. 1 provides an overview of the analytic framework used to assess cortical changes in lesion-connected regions, along with the neuroimaging markers included in the study.

### 2.7 Statistical analysis

In the direct approach, the mean value of each cortical metric within the defined ROI masks (unconnected, low-, medium-, and high-level) was used for analysis. For the indirect approach, since no standardized method exists for defining binary ROIs in ChaCo-based connectivity maps, a ChaCo-weighted averaging strategy was adopted to represent cortical properties of the lesion-connected regions.

We assessed whether regions with different connectivity levels exhibited differential rates of cortical degeneration over time using a linear mixed-effects (LME) model, with follow-up time in years, connectivity level (treated as an ordinal variable: 0 = unconnected, 1 = low-level, 2 = medium-level, 3 = high-level), and their interaction term (follow-up time × connectivity level) included as fixed effects. A random intercept was specified for each subject. Post hoc comparisons were performed to assess the rate of change across connectivity levels, treating connectivity level as a categorical factor. Resulting *p*-values were corrected for multiple comparisons using the false discovery rate (FDR) method. The same analysis was repeated using the mirrored lesion as the seed region to assess whether the differences in cortical atrophy rates could be explained by intrinsic regional vulnerability rather than by the lesion itself. In the indirect approach, longitudinal changes in cortical metrics were also assessed using LME models, with follow-up time as the independent variable and a random intercept for each subject, to determine whether cortical regions inferred to be disconnected by ChaCo weighting exhibited significant degeneration over time.

To further quantify cortical degeneration at the individual level, we calculated the percent change (pc) of each cortical metric between time points, defined as the relative change with respect to the absolute baseline value. Only the high-level connected region was selected in the direct estimation, as prior studies showed it to be the most sensitive to lesion-related cortical alterations.

To reduce dimensionality and extract key patterns of cortical change, we performed principal component analysis (PCA) on the standardized percent change values of the five cortical metrics. The suitability of the data for PCA was assessed using the Kaiser-Meyer-Olkin (KMO) measure and Bartlett’s test of sphericity. Principal components with eigenvalues greater than 1 were retained according to Kaiser’s criterion. For each subject, factor scores for the retained components were computed and used in subsequent analyses. First, to investigate whether imaging markers related to cSVD burden, white matter microstructural integrity, and lesion-related characteristics were associated with the rate of cortical degeneration, general linear models (GLM) were performed with factor scores as the dependent variable, adjusting for age, sex, and follow-up interval. In an additional model, lesion location (basal ganglia, centrum semiovale/corona radiata, or brainstem) was further included as a covariate to account for differences in affected fiber tracts due to lesion variability. All variables were standardized during this analysis to ensure comparability of the resulting regression coefficients. Second, to examine the relationship between cortical degeneration and changes in clinical outcomes, GLMs were conducted with the change in clinical scores between baseline and follow-up as the dependent variable, and factor scores as the independent variable. Covariates included age, sex, vascular risk factors, and follow-up interval. For models involving cognitive function performance, years of education were additionally included as covariates.

All statistical analyses were conducted using R (version 4.3.1). A *p*-value < 0.05 was considered statistically significant.

## 3 Results

### 3.1 Participants

From July 2018 to February 2025, 284 patients were recruited into the original RSSI cohort, of whom 132 completed the 1-year follow-up. 76 patients with RSSI were included in the final analysis (mean age at baseline: 53.91 years; 82% male), with a median follow-up interval of 1.27 years. Details of participant exclusion are presented in Fig. S1. The majority of RSSI lesions were located in the deep white matter (corona radiata: 47.37%). No participants were excluded during image analysis, as all passed quality control procedures and had successfully identified lesion-connected surface regions according to the criteria defined in the Methods section. Demographic, clinical, and imaging characteristics are presented in Table 1. Fig. S2 shows the average location of RSSI lesions in the present study in the standard space.

**Table 1:** Demographic and clinical characteristics of the study cohort at baseline and follow-up. NIHSS, National Institute of Health stroke scale; mRS, Modified Rankin Scale; MoCA, Montreal Cognitive Assessment-Beijing version; STT, Shape Trail Test; HAMA, Hamilton Anxiety Rating Scale; HAMD, Hamilton Depression Rating Scale; cSVD, cerebral small vessel disease; WMH, white matter hyperintensity; PSMD, peak width of skeletonized mean diffusivity; NAWM, normal-appearing white matter; FA, fractional anisotropy; MD, mean diffusivity.

**Variables**	**RSSI Patients (n = 76)**
Age, years, mean (SD)	53.91 (9.69)
Male sex, n (%)	62 (81.58%)
Education, years, mean (SD)	10.59 (4.76)
Hypertension, n (%)	44 (57.89%)
Diabetes, n (%)	22 (28.95%)
Hyperlipemia, n (%)	26 (34.21%)
Current Smoking, n (%)	36 (47.37%)
NIHSS, median (IQR)	3 (2 – 6)
Follow-up time, years, median (IQR)	1.27 (1.12 – 1.73)

**Lesion characteristics**	
Lesion hemisphere, left, n (%)	41 (53.95%)
Lesion diameter, cm, mean (SD)	1.68 (0.64)
Lesion location, n (%)	
Basal ganglia	30 (39.47%)
Corona radiata	36 (47.37%)
Brainstem	10 (13.16%)

**Clinical outcome**	
mRS at baseline, median (IQR)	2 (1 – 4)
mRS at follow-up, median (IQR)	1 (1 – 2)
MoCA at baseline, median (IQR)	26 (21 – 28)
MoCA at follow-up, median (IQR)	26 (22.5 – 27)
STT-A at baseline, s, mean (SD)	78.63 (41.83)
STT-A at follow-up, s, mean (SD)	68.33 (32.89)
STT-B at baseline, s, mean (SD)	177.01 (69.20)
STT-B at follow-up, s, mean (SD)	162.25 (68.20)
HAMA at baseline, median (IQR)	3 (2 – 8)
HAMA at follow-up, median (IQR)	4 (1.75 – 7)
HAMD at baseline, median (IQR)	4 (1 – 5)
HAMD at follow-up, median (IQR)	2 (1 – 5.25)

**Imaging characteristics**^a^	
cSVD score, median (IQR)	1 (0 – 2)
WMH volume (%ICV), mean (SD)	0.78 (0.42)
PSMD, 10^-3^ mm^2^ /s, mean (SD)	0.28 (0.06)
Cavitation, n (%)	58 (76.32%)
Track/Cap sign, n (%)	38 (50.00%)
NAWM FA, mean (SD)	0.41 (0.03)
NAWM MD, 10^-3^ mm^2^ /s, mean (SD)	0.76 (0.11)
Tract FA, mean (SD)	0.44 (0.04)
Tract MD, 10^-3^ mm^2^ /s, mean (SD)	0.92 (0.20)

a The cSVD total score and WMH volume were assessed at baseline, and other imaging markers were obtained at follow-up.

### 3.2 Cortical degeneration in lesion-connected region

**Fig. 2:**
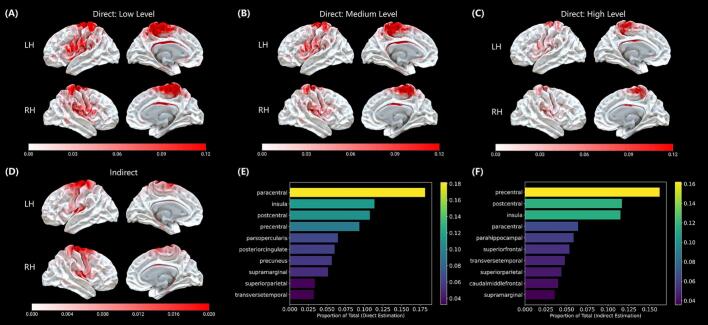
Group-level results of anatomically connected cortical regions following RSSI. (A), (B), (C): Average surface maps of three levels of lesion-connected ROIs (low, medium, and high connectivity) derived from the direct estimation approach using individual DWI data. (D): Average surface map of the indirect estimation approach based on the change in connectivity (ChaCo) values across participants. (E), (F): Top 10 cortical regions showing the highest degree of structural connectivity to the RSSI lesion, based on the Desikan-Killiany (DK) atlas. Bar plots show the proportion of each region’s average value normalized to the total sum across all regions. For direct estimation, proportions were calculated based on the tract density image, rather than binarized ROI masks. LH, left hemisphere; RH: right hemisphere.

Fig. S3 displays the average tract density image normalized by the total number of streamlines and ChaCo score across all participants in the MNI space. The corresponding lesion-connected cortical regions and ChaCo values projected onto the cortical surface are shown in Fig. 2. The connectivity maps derived from the direct and indirect approaches showed strong spatial correspondence (Fig. S4), both at the vertex level (ρ = 0.829, *p_spin_* < 0.001) and at the regional level (ρ = 0.836, *p_spin_* = 0.001). These structurally connected areas were primarily located in motor-related regions, including the paracentral, postcentral, and precentral gyri, as well as the insula cortex.

Table 2 summarizes the results of the linear mixed-effects (LME) models assessing longitudinal changes in cortical metrics. For the direct estimation, significant cortical damage related to connectivity strength was observed in both cortical thickness and grey matter volume (thickness: Time × Strength, β = -0.020, 95%CI = -0.031 – -0.009, *p* < 0.001; volume: Time × Strength, β = -0.016, 95%CI = -0.029 – -0.003, *p* = 0.013). Although pairwise comparisons between lesion-connected regions did not reach statistical significance, higher connectivity strength was associated with a steeper slope of cortical decline compared to unconnected regions, and all such comparisons were statistically significant (thickness: 0.053, 0.056, 0.065; volume: 0.042, 0.046, 0.053; all *p*_FDR_ < 0.05). Table S1 presents results from the analysis using a mirrored lesion in the contralateral hemisphere. No such trend reached statistical significance in this control analysis. The indirect approach similarly revealed significant longitudinal reductions in cortical thickness and grey matter volume within structurally connected surface regions (thickness: β = -0.019, 95%CI = -0.027 – -0.010, *p* < 0.001; volume: β = -0.017, 95%CI = -0.026 – -0.008, *p* < 0.001). A significant decline in surface area was also observed (area: β = -0.003, 95%CI = -0.006 – -1.17e-4, *p* = 0.041). The longitudinal distributions of cortical measures are shown in Fig. S5.

**Table 2:** Results of linear mixed-effects (LME) models examining RSSI-induced cortical degeneration. The first section reports the results of LME models testing whether the rate of change in cortical metrics increases with higher lesion connectivity levels (the interaction term Time × Strength), where connectivity strength was modeled as an ordinal variable (0 = unconnected, 1 – 3 = increasing levels). The second section presents post hoc pairwise comparisons of the rate of cortical change between different levels of lesion connectivity, with connectivity level modeled as a categorical factor. The third section shows whether each cortical metric changed significantly over time in the indirect estimation approach. All LME models included a random intercept for each participant.

	**Thickness**		**Volume**		**Area**		**Curv**		**Sulc**	
	Estimate (95% CI)	*p*-value	Estimate (95% CI)	*p*-value	Estimate (95% CI)	*p*-value	Estimate (95% CI)	*p*-value	Estimate (95% CI)	*p*-value
**Term (Direct Estimation)**										
Time	0.014 (-0.008 – 0.036)	0.226	0.010 (-0.015 – 0.035)	0.415	-0.004 (-0.009 – 0.002)	0.208	-0.002 (-0.005 – 0.001)	0.186	-0.093 (-0.259 – 0.072)	0.269
Strength	-0.026 (-0.043 – -0.010)	**0.002**	-0.109 (-0.128 – -0.091)	**<0.001**	-0.042 (-0.046 – -0.038)	**<0.001**	-0.002 (-0.004 – 0.001)	0.154	-0.113 (-0.235 – 0.009)	0.071
Time × Strength	-0.020 (-0.031 – -0.009)	**<0.001**	-0.016 (-0.029 – -0.003)	**0.013**	0.001 (-0.002 – 0.003)	0.699	0.002 (3.60e-4 – 0.003)	**0.015**	0.053 (-0.032 – 0.139)	0.222

**Pairwise comparison**^a^**(Direct Estimation)**										
Unconnected – Low level	0.053 (0.006 – 0.099)	**0.007**	0.042 (-0.003 – 0.087)	**0.031**	-0.002 (-0.009 – 0.005)	0.991	-0.005 (-0.011 – 0.001)	0.066	-0.080 (-0.432 – 0.271)	0.740
Unconnected – Medium level	0.056 (0.010 – 0.102)	**0.006**	0.046 (0.001 – 0.091)	**0.027**	-0.002 (-0.009 – 0.005)	0.991	-0.005 (-0.011 – 0.001)	0.066	-0.102 (-0.454 – 0.250)	0.740
Unconnected – High level	0.065 (0.019 – 0.112)	**0.002**	0.053 (0.008 – 0.098)	**0.014**	-0.002 (-0.009 – 0.005)	0.991	-0.006 (-0.012 – 1.04e-4)	0.066	-0.170 (-0.522 – 0.181)	0.740
Low level – Medium level	0.003 (-0.043 – 0.049)	0.866	0.003 (-0.042 – 0.048)	0.847	-6.58e-5 (-0.007 – 0.007)	0.991	-3.32e-4 (-0.006 – 0.006)	0.887	-0.021 (-0.373 – 0.330)	0.875
Low level – High level	0.013 (-0.034 – 0.059)	0.712	0.011 (-0.034 – 0.056)	0.790	-3.21e-5 (-0.007 – 0.007)	0.991	-0.001 (-0.007 – 0.005)	0.887	-0.090 (-0.441 – 0.262)	0.740
Medium level – High level	0.010 (-0.037 – 0.056)	0.712	0.008 (-0.037 – 0.053)	0.791	3.37e-5 (-0.007 – 0.007)	0.991	-0.001 (-0.007 – 0.005)	0.887	-0.068 (-0.420 – 0.283)	0.740

**Term (Indirect Estimation)**										
Time	-0.019 (-0.027 – -0.010)	<0.001	-0.017 (-0.026 – -0.008)	<0.001	-0.003 (-0.006 – -1.17e-4)	0.041	-4.33e-4 (-8.68e-4 – 1.68e-6)	0.051	-0.001 (-0.022 – 0.019)	0.896

a : *p*-values in the pairwise comparisons were FDR-corrected.

### 3.3 Principal component analysis

The suitability of the data for PCA is described in the Supplementary Materials. Results of the PCA showed similar patterns between the direct and indirect estimations. The first two components (PC1 and PC2) had standard deviations greater than 1 and were therefore retained for subsequent analyses (direct estimation: 2.31 and 1.06; indirect estimation: 2.10 and 1.00).

**Fig. 3:**
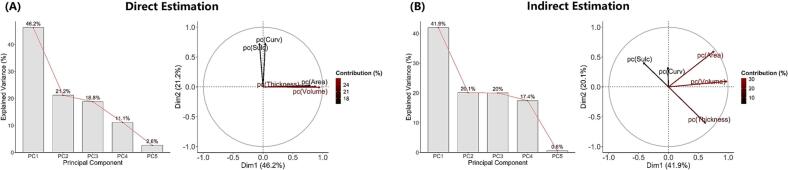
Results of principal component analysis (PCA). (A) Direct estimation; (B) Indirect estimation. Left panels: Scree plots showing the percentage of variance explained by each principal component (PC). Components with eigenvalues > 1 were retained for further analysis. Right panels: Variable factor maps displaying the contribution and direction of each cortical metric to the first two principal components (PC1 and PC2). Arrows represent the projection of each metric in the two-dimensional component space; arrow length and color intensity reflects the strength of contribution, and direction indicates the sign of loading. pc, percent change of each cortical metric.

Fig. 3 presents the scree plot and variable factor map, showing the proportion of variance explained by each component and the contribution of individual cortical measures to PC1 and PC2. In both approaches, PC1 was characterized by features of cortical parenchymal atrophy, with all factor loadings for volume, thickness, and surface area exceeding 0.4. In the direct estimation, PC2 primarily reflected geometric alterations of the cortex, with strong loadings from sulcal depth and curvature (both > 0.7). In contrast, PC2 in the indirect estimation also received moderate contributions from cortical thickness and surface area in addition to sulcal depth and mean curvature. PC1 and PC2 were deemed acceptable for further analysis, as they captured the two distinct dimensions of cortical change: parenchymal atrophy and surface morphological alterations. For interpretability, lower PC1 scores corresponded to greater reductions in cortical thickness, volume, and surface area, indicating more pronounced cortical atrophy, while larger absolute PC2 values reflected greater magnitudes of cortical morphological change.

### 3.4 Relationship between imaging markers and cortical degeneration

**Fig. 4:**
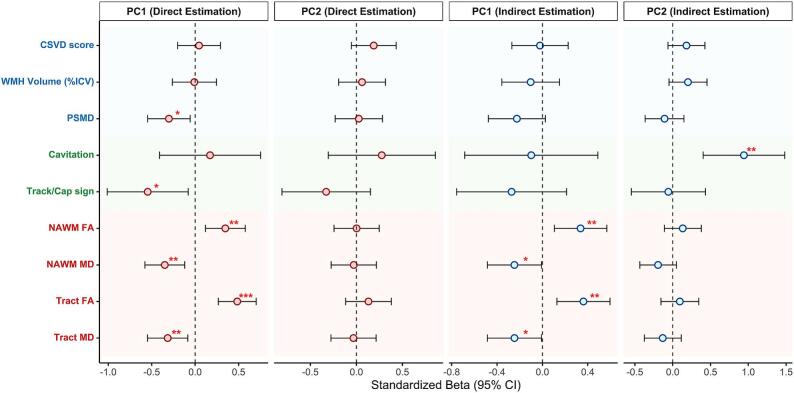
Relationship between imaging markers and cortical alterations. Standardized beta coefficients and 95% confidence intervals from regression analyses are shown for each imaging marker in relation to the two principal components (PC1 and PC2). Imaging markers are classified according to their category (blue: cSVD imaging markers; green: RSSI lesion evolution; red: DTI metrics). cSVD, cerebral small vessel disease; DTI, diffusion tensor imaging; RSSI, recent small subcortical infarction; WMH, white matter hyperintensity; ICV, intracranial volume; PSMD, peak width of skeletonized mean diffusivity; NAWM, normal-appearing white matter; FA, fractional anisotropy; MD, mean diffusivity. *: *p* < 0.05; **: *p* < 0.01; ***: *p* < 0.001. (For interpretation of the references to colour in this figure legend, the reader is referred to the web version of this article.)

The forest plot (Fig. 4) illustrates the associations between MRI imaging markers and the principal components of cortical degeneration. For PC1, which reflected cortical atrophy, DTI metrics (FA and MD) within NAWM and lesion-connected tracts were significantly associated with the component scores in both the direct (NAWM FA: β = 0.35, 95%CI = 0.12 – 0.58, *p* = 0.004; NAWM MD: β = -0.35, 95%CI = -0.58 – -0.12, *p* = 0.004; Tract FA: β = 0.48, 95%CI = 0.27 – 0.70, *p* < 0.001; Tract MD: β = -0.32, 95%CI = -0.55 – -0.08, *p* = 0.010) and indirect approaches (NAWM FA: β = 0.34, 95%CI = 0.10 – 0.57, *p* = 0.006; NAWM MD: β = -0.25, 95%CI = -0.49 – -0.01, *p* = 0.047; Tract FA: β = 0.36, 95%CI = 0.13 – 0.60, *p* = 0.004; Tract MD: β = -0.25, 95%CI = -0.49 – -0.01, *p* = 0.047). After excluding lesion-connected white matter regions, diffusion metrics derived from ncNAWM remained highly correlated with those from NAWM (FA: r = 0.98, *p* < 0.001; MD: r > 0.99, *p* < 0.001), reflecting the relatively small proportion of lesion-connected tracts within the overall white matter mask. Regression analyses using ncNAWM metrics yielded results comparable to those obtained with NAWM (Table S2). Additionally, the presence of the track/cap sign and PSMD was also found to be related to focal cortical degeneration (PSMD: β = -0.30, 95%CI = -0.55 – -0.06, *p* = 0.018, track/cap sign: β = -0.55, 95%CI = -1.01 – -0.08, *p* = 0.025) under direct estimation. In the indirect approach, cavitation of the RSSI lesion was positively associated with the PC2 factor score (β = 0.94, 95%CI = 0.40 – 1.48, *p* = 0.001). Fig. S6 shows the results of the regression model, additionally adjusting for infarct location, which demonstrated similar findings.

### 3.5 Cortical degeneration and clinical outcomes

**Fig. 5:**
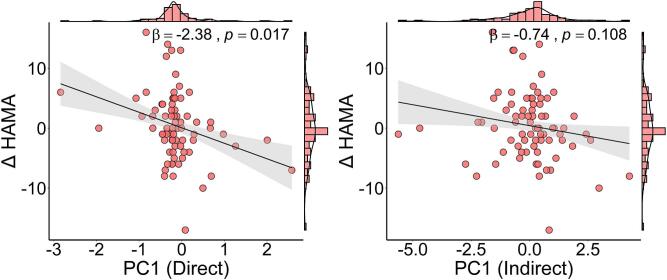
Association between cortical degeneration and the change in HAMA scores. Scatter plots illustrate the relationship between the first principal component (PC1), reflecting cortical atrophy, and the change in HAMA scores from baseline to follow-up, using the direct (left) and indirect (right) approaches. Regression models were adjusted for age, sex, years of education, VRF score, and follow-up time interval. HAMA, Hamilton Anxiety Rating Scale; VRF, Vascular Risk Factor.

In the direct estimation, a faster rate of cortical atrophy was significantly associated with a greater increase in HAMA scores (Fig. 5, β = -2.38, 95%CI = -4.30 – -0.47, *p* = 0.017). A similar trend was observed in the indirect estimation, though it did not reach statistical significance (Fig. 5, β = -0.74, 95%CI = -1.64 – -0.15, *p* = 0.108). Table S3 presents the full regression results. The PC2 score, which reflects cortical morphological changes, was significantly associated with changes in STT-A completion time (PC2 from direct estimation: β = 12.94, 95%CI = 5.50 – 20.38, *p* = 0.001; PC2 from indirect estimation: β = 9.47, 95%CI = 1.56 – 17.39, *p* = 0.022).

## 4 Discussion

The present study focused on secondary damage in cortical regions resulting from the remote effects of RSSI, and compared results from patient-specific DWI data at follow-up with those derived from a normative connectome of healthy individuals. Our findings demonstrate focal cortical atrophy driven by lesion-associated structural connectivity, which is associated with both visual imaging features and quantitative DTI metrics. Moreover, the observed pattern of cortical changes paralleled alterations in anxiety levels and executive function.

Lesion-connected cortical regions exhibited a faster rate of change in both cortical thickness and gray matter volume compared to unconnected regions. As anticipated, regions with stronger connectivity showed more pronounced alterations (Wei et al., 2019), evidenced by a stepwise trend in effect sizes during pairwise comparisons with unconnected regions. However, post hoc comparisons among the connected regions did not reach statistical significance. This may be partly attributable to the methodological design of the connectivity-level ROIs, which are hierarchically nested rather than mutually exclusive. Additionally, the modest sample size and limited follow-up duration, compared to other longitudinal studies of cortical alterations (Bernal et al., 2024), may have reduced the statistical power to detect more pronounced cortical degeneration. Notably, when the mirrored lesion ROI was used as the seed for tractography, no accelerated cortical thinning was observed in the connected regions. This supports the hypothesis that the observed cortical degeneration was primarily driven by lesion-specific structural connectivity rather than by inherent regional vulnerability to age-related atrophy.

We found that imaging markers associated with white matter injury, including the presence of the track/cap sign, reduced FA, and increased MD in both NAWM and lesion-connected tracts, were significantly correlated with progression of cortical atrophy identified by PCA. Recognized to reflect white matter integrity, DTI measures like FA in cSVD patients show significant differences from healthy controls at the whole-brain level and are established cSVD markers (Liu et al., 2020). The track/cap sign, a recently described imaging marker following RSSI, has also been associated with poorer functional outcomes and the small vessel disease progression (Cheng et al., 2025, Wang et al., 2020). Our findings suggest that the track/cap sign may serve as a readily identifiable radiological marker of secondary degeneration following RSSI, potentially sharing pathophysiological mechanisms with DTI metrics. In contrast, infarct cavitation was not correlated with accelerated cortical atrophy, which is similar to findings from Duering et al. on subcortical ischemic lesions of different etiologies, further supporting the notion that lesion evolution may not directly drive perilesional degeneration (Loos et al., 2018). Moreover, a higher baseline cSVD total score and WMH volume were not associated with more severe focal cortical degeneration. Although prior studies have established associations between WMH and cortical thinning across various lobes (Bernal et al., 2024, Li et al., 2023), such widespread effects might be overshadowed by the localized impact of RSSI lesions. Taken together, our results suggest that RSSI could induce neuronal necrosis and subsequent Wallerian degeneration, leading to distal axonal injury and cortical atrophy in structurally connected regions. However, the observed associations between cortical atrophy and both tract-specific and ncNAWM diffusion metrics, along with PSMD, suggest that diffuse white matter integrity may also influence the extent of remote cortical changes. Future studies incorporating longitudinal diffusion MRI are warranted to disentangle these mechanisms and clarify their temporal relationships.

Cortical degeneration in lesion-connected regions was found to parallel increases in HAMA scores, indicating a possible link between structural disconnection and anxiety symptoms in RSSI. We speculate that insular cortex atrophy may be a primary contributor, as supported by numerous neuroimaging studies involving anxiety disorders (Laird et al., 2019). Furthermore, studies of brain connection have demonstrated that altered connectivity between the insula and other key regions involved in anxiety circuits, such as the amygdala, may underlie elevated anxiety symptoms (Baur et al., 2013). In addition, the principal component reflecting cortical morphological changes was associated with executive function performance. However, it is important to note that the shape metrics include both positive and negative values across sulcal and gyral regions, and the anatomical heterogeneity of lesion-connected cortical areas complicates the interpretation of their relationship with cognitive outcomes. Several factors may account for the absence of a strong association between RSSI-related cortical atrophy and changes in global cognitive performance. The present study focused on localized cortical structural alterations, which vary across individuals depending on infarct location and their structural connectome. Such focal changes may be less sensitive to global cognitive decline compared with markers reflecting overall brain integrity, such as global brain atrophy (Hassani et al., 2025). Second, prior studies have highlighted the importance of frontal lobe in cognitive performance, and recent evidence suggests that the prefrontal cortex may serve as a central hub of structural diaschisis following subcortical stroke (Li et al., 2026). In our analysis, however, the lesion-connected cortical regions did not predominantly overlap with frontal areas, and we did not directly assess widespread network disruption, which may better capture cognitive vulnerability (Sagnier et al., 2017). Besides, cognitive outcomes were assessed based on changes over a relatively short follow-up interval. It is possible that cognitive alterations occurred in the acute or subacute phase prior to baseline assessment, thereby attenuating detectable longitudinal associations. Longer follow-up and domain-specific cognitive assessments are needed to clarify the temporal relationship between structural disconnection and cognitive decline.

In the present study, both the direct and indirect approaches yielded convergent results despite employing distinct methods to define the extent of cortical involvement by the infarction. This supports that the indirect approach could serve as a practical method to capture lesion-induced disconnection, as it does not require the acquisition of individual diffusion MRI data. Meanwhile, metrics from the patient-specific DWI dataset showed stronger relationships with individual imaging features and behavioral outcomes, suggesting that it might more accurately reflect subject-specific characteristics. This difference may partly stem from population disparities between our Asian cohort and the predominantly Western HCP dataset, as well as potential imaging registration inaccuracies affecting the indirect estimations (Glasser et al., 2016). Nevertheless, both methods rely on streamline counts connecting the lesion to cortical regions as a key indicator of lesion impact. Given that microstructural alterations in white matter were also related to cortical atrophy, this highlights a potential limitation of streamline count-based approaches for assessing diaschisis (Carrera and Tononi, 2014, Salvalaggio et al., 2020). In RSSI, where the underlying pathology involves infarction and necrosis rather than complete fiber transection, streamline counts alone may not fully capture the extent of disconnection. Future research may benefit from incorporating additional tract-level measures of tissue integrity beyond merely the streamline count to better characterize remote cortical damage from subcortical ischemic lesions.

We acknowledge several limitations of the present study. First, the diffusion MRI was acquired using a single-shell DWI protocol with limited angular resolution. This may affect the accuracy of probabilistic tractography, particularly in regions with complex fiber configurations. In addition, the single-shell acquisition precluded the estimation of advanced microstructural models (e.g., neurite orientation dispersion and density imaging), thereby limiting our ability to characterize white matter microstructure beyond conventional tensor-derived metrics (Petersen et al., 2024). Second, lesion masks were delineated at baseline, while follow-up DWI data were used to define the cortical regions structurally connected to the lesion. This mismatch could introduce inaccuracies due to possible post-RSSI fiber reorganization and does not capture the dynamic evolution of lesion-related connectivity over time. Moreover, given that both supratentorial and infratentorial lesions were included in the present study, the affected fiber differs accordingly. Infratentorial lesions predominantly involve projection fibers, whereas supratentorial subcortical infarcts may disrupt a broader range of white matter pathways, including long-range association fibers. This anatomical variability may introduce heterogeneity when comparing lesion-connected tracts and their connected cortical regions. Although we included infarct location as a covariate, this cannot fully account for such variability. Finally, we focused on focal lesion-to-cortex disconnection patterns rather than broader network-level alterations, which have been shown to predict post-stroke prognosis effectively (Ríos et al., 2025). This may limit the predictive utility of our approach for functional outcomes. Nevertheless, the remote effects we examined are anatomically well-defined, providing insight into studying lesion-symptom relationships in RSSI.

In conclusion, by combining longitudinal structural MRI with both direct and indirect analytical strategies, our study validated RSSI-related secondary cortical damage and identified its imaging correlates and clinical relevance. Future studies in larger cohorts are needed to confirm these findings and determine their predictive value for long-term prognosis in RSSI patients.

## Research ethics and informed consent

The Medical Ethics Committee of West China Hospital, Sichuan University, approved this study (No. 2018521). Informed consent was provided by all subjects enrolled in the study.

## Sources of funding

This work is supported by the 10.13039/501100001809National Natural Science Foundation of China (No. 82271328, 82371322, and 82301661), Sichuan Science and Technology Program (No.2024YFFK0314), Post-Doctor Research Project, 10.13039/501100013365West China Hospital, Sichuan University (No. 2023HXBH007; No. 2024HXBH041), and the Joint Funds of the 10.13039/501100001809National Natural Science Foundation of China (No. U24A20690).

## CRediT authorship contribution statement

**Youjie Wang:** Writing – review & editing, Writing – original draft, Visualization, Software, Methodology, Formal analysis, Conceptualization. **Jingyu Cui:** Investigation, Data curation. **Yuying Yan:** Investigation, Formal analysis, Data curation. **Tang Yang:** Investigation, Data curation. **Yue Yuan:** Investigation, Formal analysis, Data curation. **Rumei Lei:** Investigation, Data curation. **Rongfeng Luo:** Investigation, Data curation. **Bo Wu:** Writing – review & editing, Supervision, Resources, Conceptualization. **Shuai Jiang:** Writing – review & editing, Supervision, Project administration, Investigation, Conceptualization.

## Declaration of competing interest

The authors declare that they have no known competing financial interests or personal relationships that could have appeared to influence the work reported in this paper.

## Data Availability

Data will be made available on request. Mr. Wang had full access to all the data in the study and took full responsibility for the integrity of the data and the accuracy of the analyses. The neuroimaging analysis code is available at https://github.com/LuuuXG/cvdproc. The source data is available from the corresponding author upon reasonable request and subject to institutional and ethical regulations.
