# Supplementary material for: Remote cortical degeneration related to structural connectivity following recent small subcortical infarcts
Source: Neuroimage Clin. 2026 Mar 20;50:103987. doi: 10.1016/j.nicl.2026.103987 (PMC13049619; doi:10.1016/j.nicl.2026.103987)
Supplement: Supplementary Data 2 [file mmc2.docx]

**Supplementary Materials**

**Manuscript title:** Remote Cortical Degeneration Related to Structural Connectivity Following Recent Small Subcortical Infarcts

**Contents:**

1. Additional methodological details regarding MRI processing
2. Evaluation of Data Suitability for Principal Component Analysis
3. Figure S1: Flowchart of Patient Selection and Exclusion
4. Figure S2: Visualization of Average Lesion Distribution across Participants
5. Figure S3: Averaged Tract Density Image and Change in Connectivity (ChaCo) Maps Across Subjects
6. Figure S4: Comparison Between Connectivity Maps from Direct and Indirect Approaches
7. Figure S5: Changes in Cortical Metrics of Direction Estimation and Indirect Estimation
8. Figure S6: Relationship between Neuroimaging Markers and Cortical Alterations after Adjusting for Lesion Location
9. Table S1: Results of Cortical Alterations Using the Contralateral Mirror Lesion Masks
10. Table S2: Diffusion Metrics from Different White Matter Regions and Their Associations with Principal Components of Cortical Degeneration
11. Table S3: Association between Cortical Degeneration and Clinical Outcomes in RSSI
12. **Additional methodological details regarding MRI processing**

We used the WMH-SynthSeg tool to perform white matter hyperintensity (WMH) segmentation, as its applicability had been validated in T2 fluid attenuated inversion recovery (FLAIR) data from our center (Wang et al., 2025). Moreover, WMH-SynthSeg provides WMH segmentation results in 1 mm isotropic voxels, which facilitates accurate transformation into diffusion space. WMHs were not further categorized into periventricular and deep subtypes due to the limited resolution of the T2 FLAIR images, which constrained the detection of small lesions in the deep white matter. Baseline WMH burden was used in the present study, as (1) the limited spatial resolution prevented accurate quantification of subtle WMH progression over time (Lin et al., 2025), and (2) segmentations at follow-up were potentially confounded by evolving infarct lesions that could mimic WMH characteristics.

Peak width of skeletonized mean diffusivity (PSMD) is a diffusion-based imaging marker that has been validated across large-scale cohorts as a robust indicator of white matter injury and a sensitive surrogate of CSVD burden (Zanon Zotin et al., 2023). Higher PSMD values are considered to reflect greater microvascular damage. In this study, PSMD was calculated from the preprocessed DTI data using an open-source pipeline (<https://github.com/miac-research/psmd>), with lesion masks applied as exclusion regions to avoid confounding by focal pathology.

To obtain diffusion metrics (i.e., FA and MD) within both normal-appearing white matter (NAWM) and lesion-connected tracts at follow-up, preprocessed DTI data were first fitted with a diffusion tensor model using FSL. Each participant’s white matter mask was combined from FreeSurfer segmentations, including cerebral white matter (labels 2 and 41), cerebellar white matter (labels 7 and 46), white matter hypointensities (label 77), optic chiasm (label 85), and corpus callosum (labels 251–255). These masks were transformed into the native DTI space using affine registration from T1-weighted to FA images. The NAWM mask was then generated by subtracting the RSSI lesion mask and the WMH mask from the overall white matter mask. Mean FA and MD values within the resulting NAWM mask were calculated for analysis. To map DTI metrics from lesion-connected tracts, probabilistic tractography was performed using the lesion mask as the seed region, retaining 10,000 streamlines per participant. At each tracking step, a diffusion tensor model was fitted to a bootstrapped realization of the local DTI data, and streamlines were propagated along the direction of the principal eigenvector (Jones, 2008). Mean FA and MD values were calculated along each streamline using a precise voxel-wise mapping approach (Smith et al., 2013) and then averaged across all streamlines to yield tract-level FA and MD values for the lesion-connected fibers. Given our hypothesis that lesion-connected white matter regions may undergo secondary microstructural alterations, and that these regions may be included within the conventional NAWM definition, we additionally defined non-connected normal-appearing white matter (ncNAWM). To achieve this, a tract density image (TDI) was generated in volumetric space from the probabilistic tractography results and normalized by the total number of generated streamlines (10,000 per participant). A threshold of 2 × 10⁻⁴, as previously described (Li et al., 2023), was applied to binarize the tract density map and define the lesion-connected white matter region. The binarized mask was then subtracted from the NAWM mask to obtain the ncNAWM mask. Mean FA and MD values within the ncNAWM were subsequently calculated.

The direct estimation of lesion-related cortical surface was performed using FSL’s FMRIB’s Diffusion Toolbox (FDT). Specifically, probabilistic diffusion modeling was performed using the ‘bedpostx’ command with default parameters (two fibres per voxel and monoexponential model for single-shell DTI data). Subsequently, probabilistic tractography was carried out using the ‘probtrackx2’ command. Prior to it, we computed the affine transformation between the individual diffusion space (FA map) and Freesurfer structural space via linear registration. This affine was applied to the RSSI lesion mask in diffusion space to transform it into Freesurfer volume space, where it was used as the seed mask for tractography. To constrain streamlines to the cortex, a binary cortical gray matter mask was created by combining the Freesurfer cortical masks (*h.ribbon.mgz) from both hemispheres and used as waypoint masks in tractography. For each subject, 10,000 streamlines were sampled per seed voxel. All other parameters were set to default (number of steps per sample = 2000; step length = 0.5 mm; curvature threshold = 0.2). The resulting streamline density map (fdt_paths.nii.gz) was projected onto the subject’s cortical surface using Freesurfer’s ‘mri_vol2surf’ command and registration files from the recon-all workflow. Surface tract density maps from both hemispheres were merged, normalized by the total number of streamlines (number of seed voxels × 10,000), and non-cortical regions (e.g., medial wall) were excluded. A threshold of 3.8 × 10⁻⁵, adopted from previous literature (Duering et al., 2012; Li et al., 2023), was used to define the minimum value for connected cortical vertices. Vertices below this threshold were classified as unconnected regions. Among connected vertices, the top 50% were defined as medium-level connected regions, and the top 25% as high-level connected regions. To ensure that cortical atrophy in lesion-connected areas was not solely attributable to regional vulnerability, the same tractography procedure was repeated using a contralateral mirror lesion mask as the seed, with all other parameters unchanged (Duering et al., 2015).

In addition, we implemented an indirect estimation approach using normative connectivity from the Human Connectome Project (HCP), which does not rely on individual diffusion data. Specifically, we used the Network Modification (NeMo) toolbox (<https://github.com/kjamison/nemo>), which computes voxel-wise Change in Connectivity (ChaCo) scores to quantify lesion-induced disconnection (Kuceyeski et al., 2014). The reference connectome was built from diffusion and structural MRI data of 420 healthy subjects via the HCP minimal processing pipeline, followed by tractography using the probabilistic iFOD2 algorithm with anatomically constrained tractography (ACT). Each lesion mask, registered to MNI space, was input into the NeMo pipeline to generate a 1 mm resolution ChaCo map, defined as the proportion of lesion-intersecting streamlines terminating in each voxel. ChaCo maps were averaged across the 420 subjects and smoothed with a 6 mm Full-Width-Half-Max (FWHM) kernel, which improves inter-subject overlap. The averaged ChaCo maps were then projected to the 164k ‘fsaverage’ cortical surface using a precomputed transformation matrix from the ‘neuromaps’ toolbox ([https://github.com/netneurolab/neuromaps](https://github.com/netneurolab/neuromaps?utm_source=chatgpt.com)), yielding a ChaCo value for each cortical vertex.

All MRI processing codes, including the direct and indirect estimation methods, can be found at <https://github.com/LuuuXG/cvdproc>.

1. **Evaluation of Data Suitability for Principal Component Analysis**

For the percent change in five cortical measures derived from the direct estimation, the overall Kaiser-Meyer-Olkin (KMO) value was 0.58, indicating a mediocre level of shared variance among the variables. Bartlett’s test (χ^2^(10) = 128.82, *p* < 0.001) further supported the presence of adequate inter-variable correlations for performing PCA. The KMO value was 0.29 for indirect estimation, and Bartlett’s test yielded χ^2^(10) = 214.39, *p* < 0.001. Although the KMO value was below the conventional threshold (< 0.5), an exploratory PCA was still performed to maintain consistency across analytical strategies.

1. **Flowchart of Patient Selection and Inclusion**


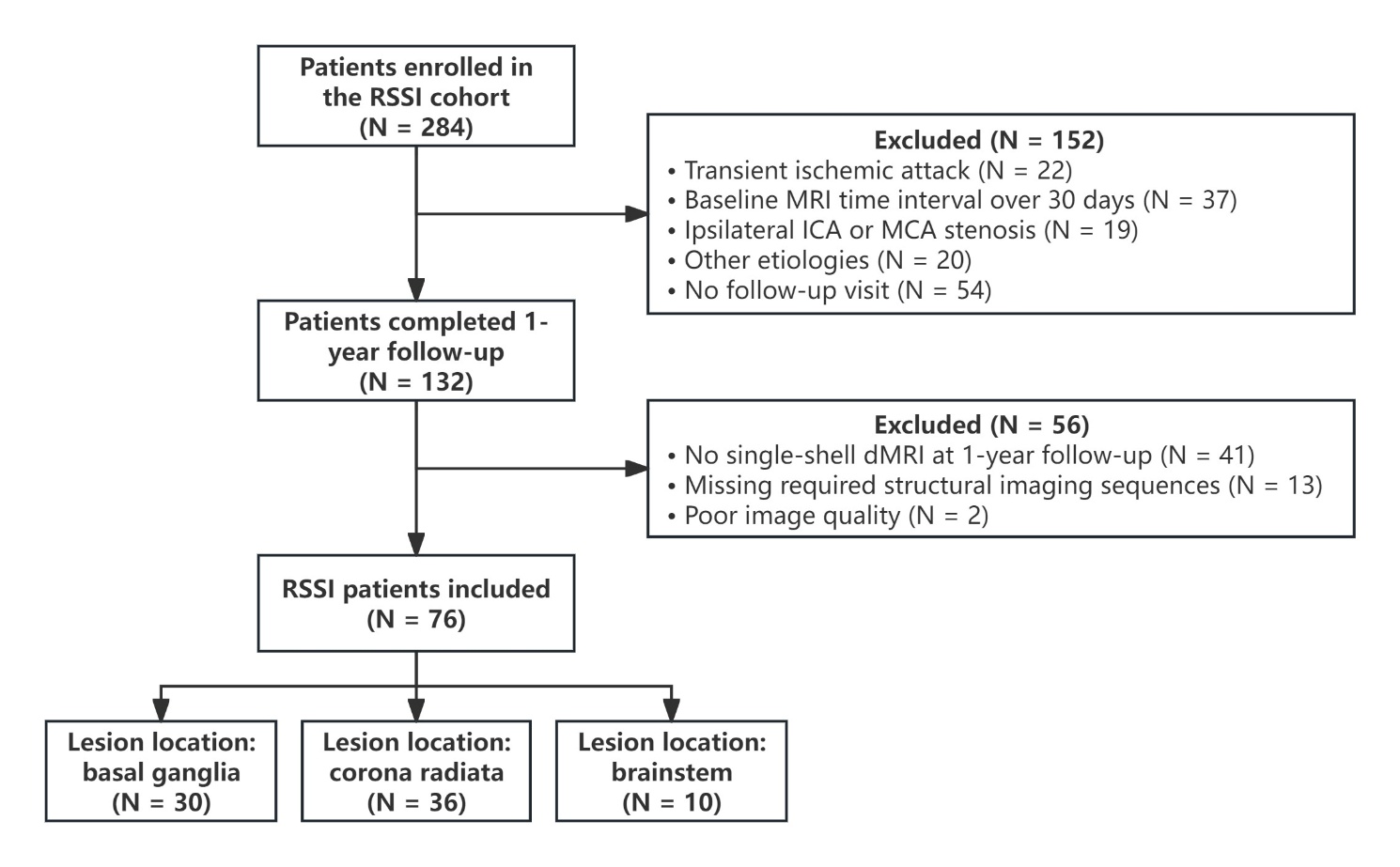


**Figure S1.** From July 2018 to February 2025, 284 patients were enrolled in the original RSSI cohort. After applying baseline exclusion criteria and follow-up availability, 132 patients completed the 1-year follow-up. Of these, 56 were further excluded due to absence of single-shell diffusion MRI at follow-up, missing required structural imaging sequences, or poor image quality. A total of 76 RSSI patients were included in the final analysis.

1. **Visualization of Average Lesion Distribution across Participants**


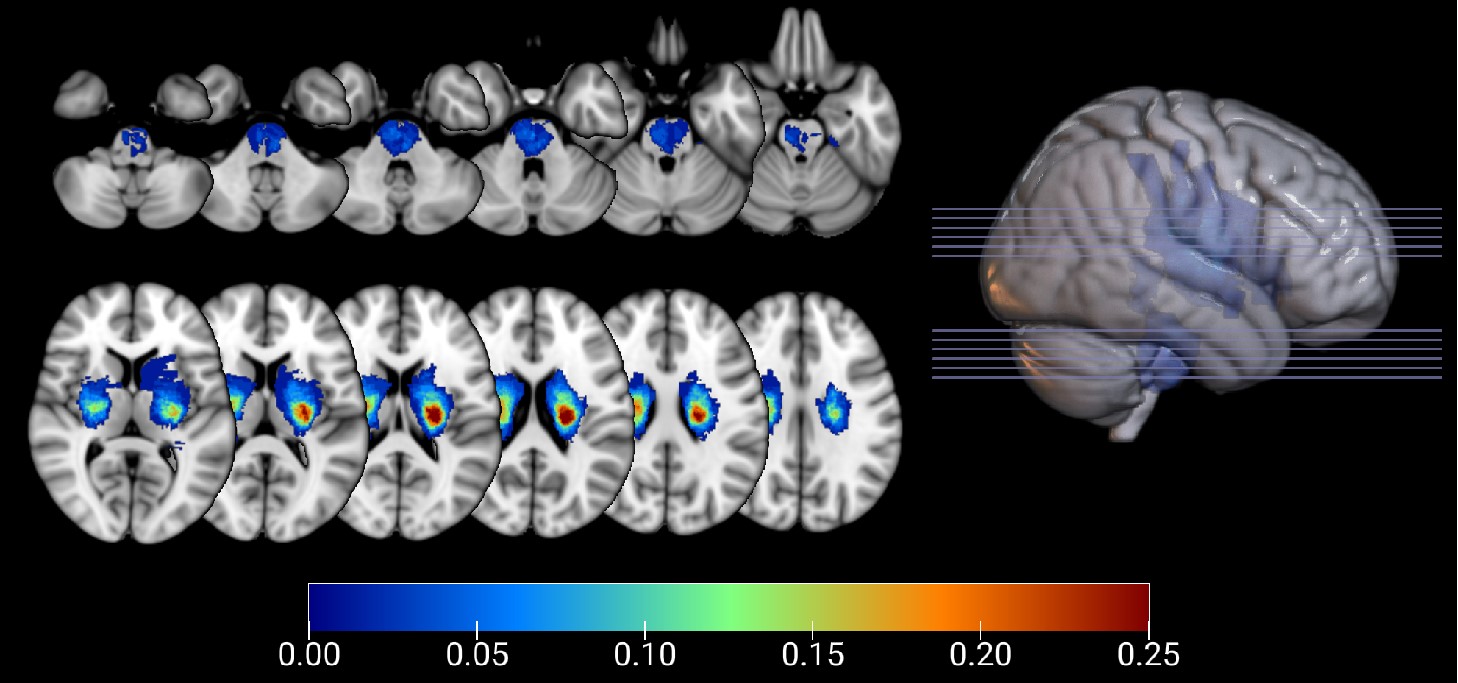


**Figure S2.** The figure presents the average location of RSSI lesions in the cohort in the MNI space. The color scale indicates the proportion of participants with lesions in each voxel, with warmer colors representing higher overlap across participants.

1. **Averaged Tract Density Image and Change in Connectivity (ChaCo) Maps Across Subjects**


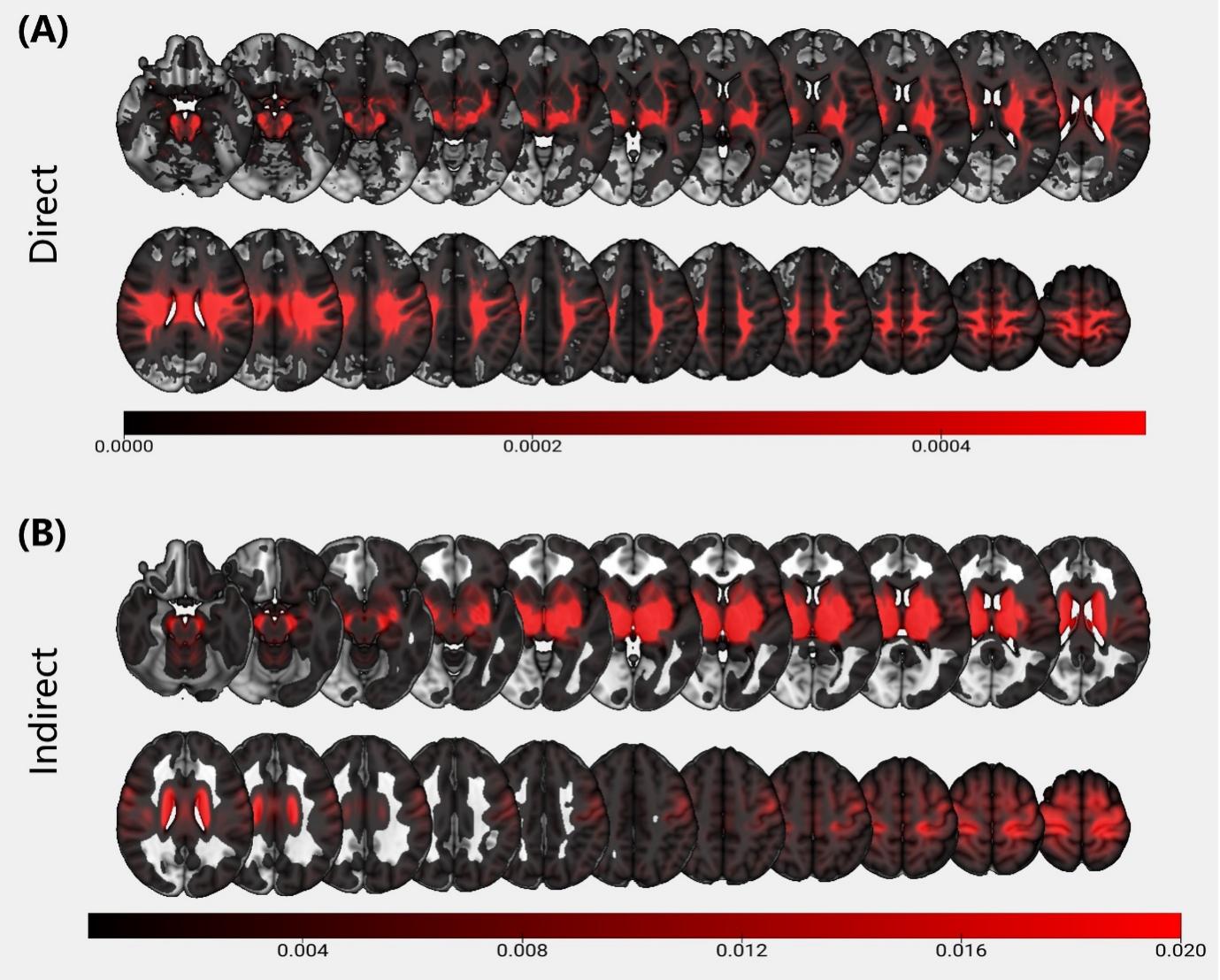


**Figure S3.** Group-averaged tract density image and change in connectivity (ChaCo) maps in standard MNI space.

1. **Comparison Between Connectivity Maps from Direct and Indirect Approaches**


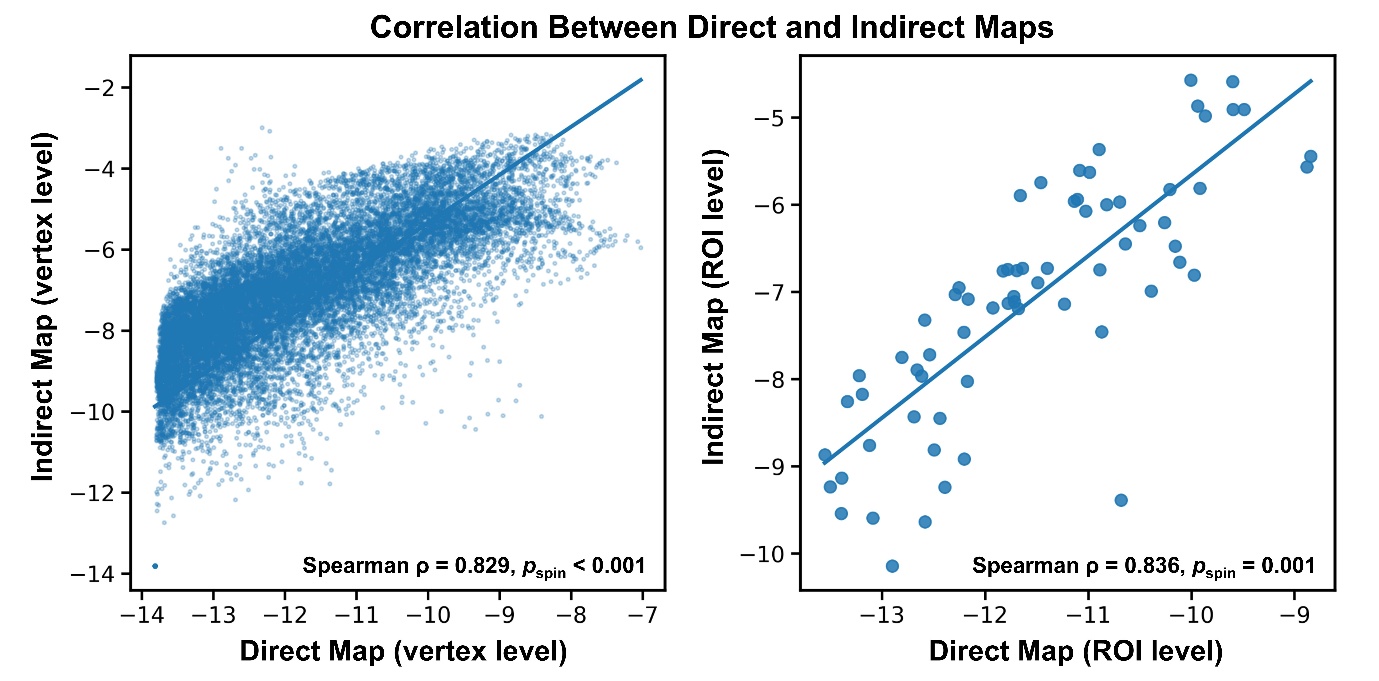


**Figure S4.** Spatial correspondence between connectivity maps derived from the direct and indirect approaches. Left panel: Vertex-wise correlation; Right panel: Regional-level correlation. Both maps were averaged across participants and log-transformed prior to analysis. Spearman’s rank correlations were calculated, and statistical significance was assessed using spatially constrained spin tests with 5,000 permutations.

1. **Changes in Cortical Metrics of Direction Estimation and Indirect Estimation**


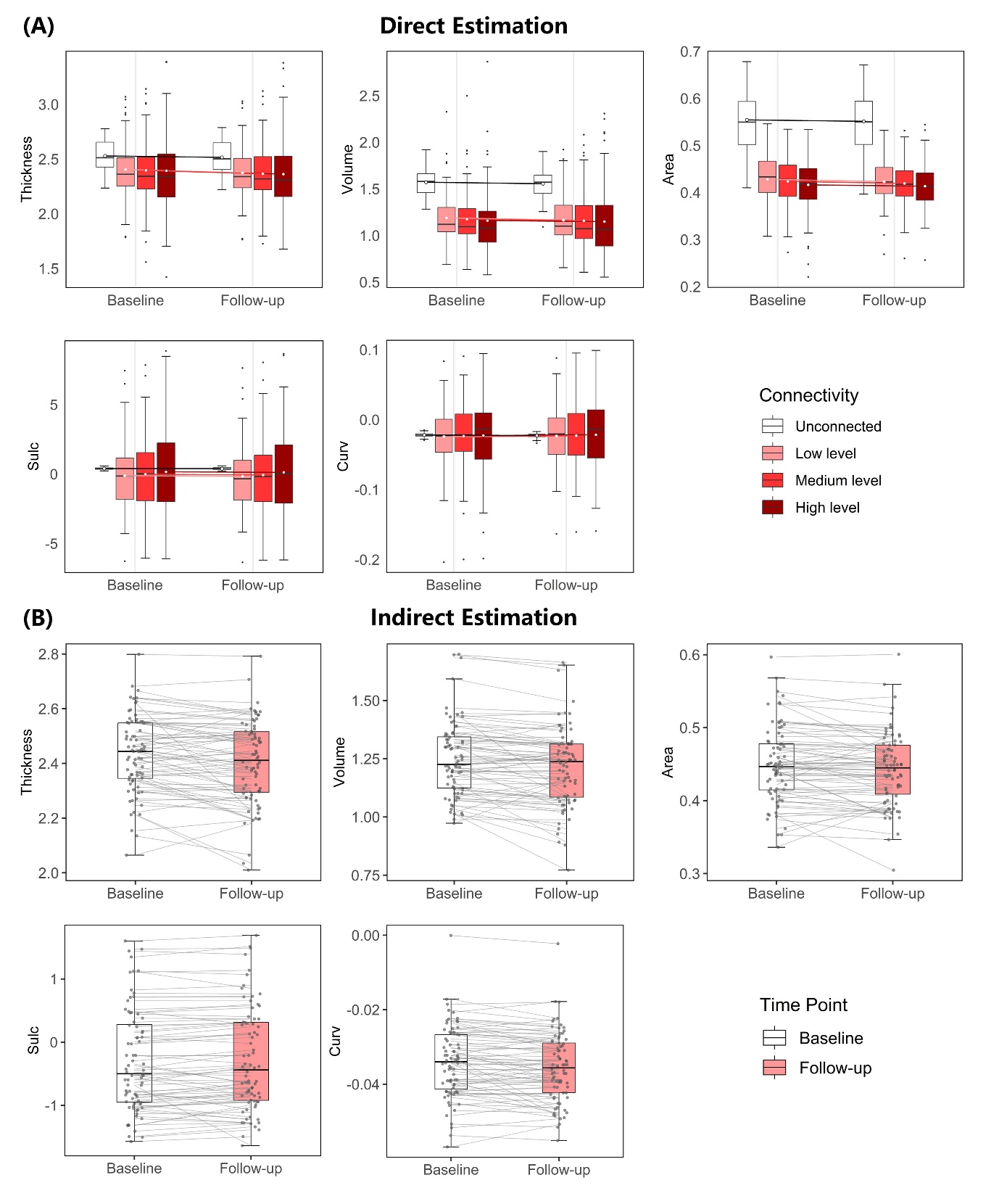


**Figure S5.** Box plots illustrating the longitudinal changes in cortical measures. (A): Direct estimation. Box colors indicate different connectivity strengths, and lines represent the change in the mean values between baseline and follow-up. (B): Indirect estimation. Lines indicate individual-level changes in cortical measures between time points.

1. **Relationship between Neuroimaging Markers and Cortical Alterations after Adjusting for Lesion Location**


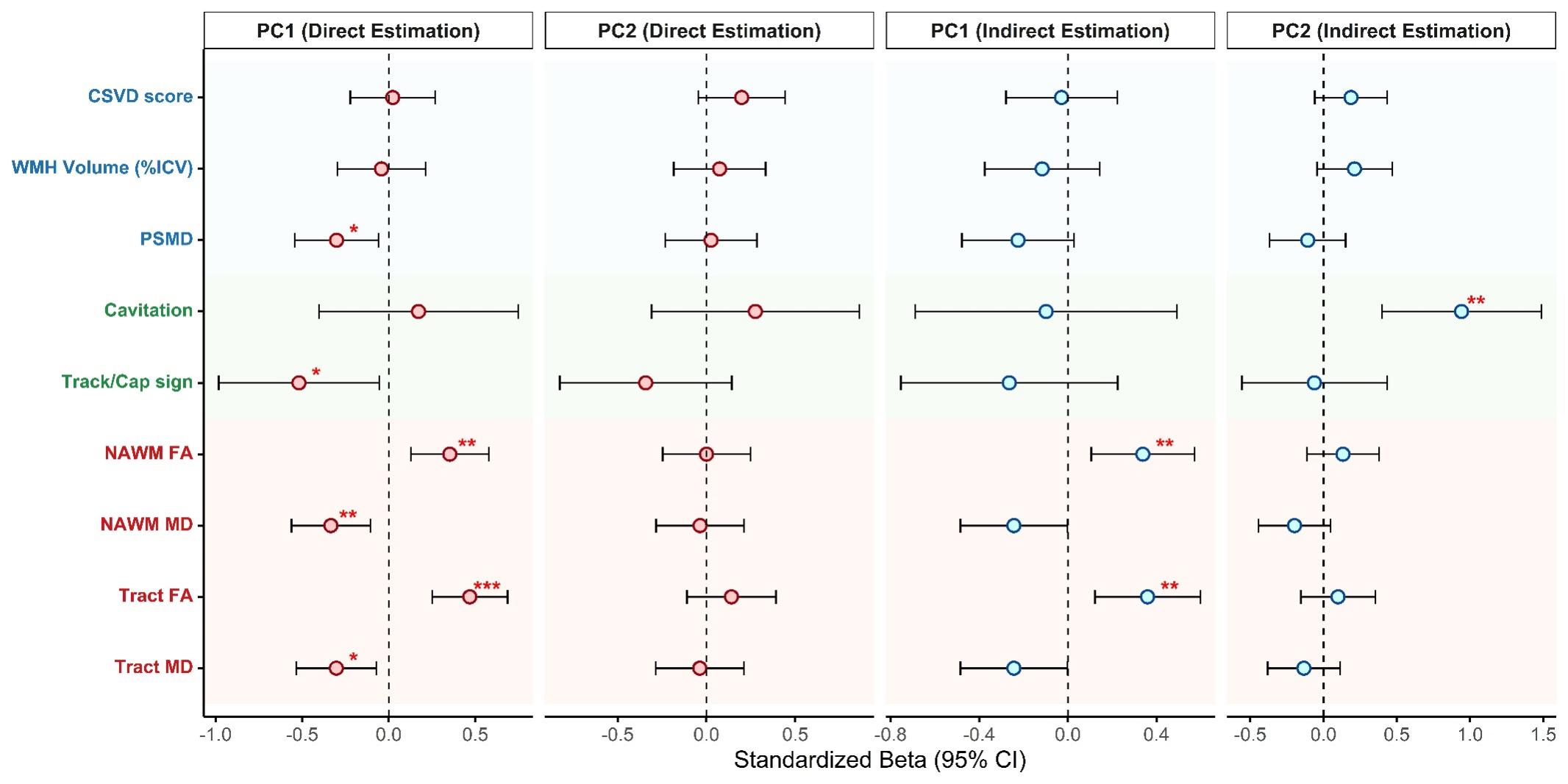


**Figure S6.** Relationship between imaging markers and principal component factor values. Forest plots display standardized beta coefficients and 95% confidence intervals. Age, sex, follow-up interval, and lesion location were included as covariates in all models.

CSVD, cerebral small vessel disease; WMH, white matter hyperintensity; ICV, intracranial volume; PSMD, peak width of skeletonized mean diffusivity; NAWM, normal-appearing white matter; FA, fractional anisotropy; MD, mean diffusivity.

*: *p* < 0.05; **: *p* < 0.01; ***: *p* < 0.001.

**9. Results of Cortical Alterations Using the Contralateral Mirror Lesion Masks**

|  | **Thickness** | | **Volume** | | **Area** | | **Curv** | | **Sulc** | |
| --- | --- | --- | --- | --- | --- | --- | --- | --- | --- | --- |
|  | Estimate (95% CI) | *p*-value | Estimate (95% CI) | *p*-value | Estimate (95% CI) | *p*-value | Estimate (95% CI) | *p*-value | Estimate (95% CI) | *p*-value |
| **Term (Direct Estimation)** | | | | | | | | | | |
| Time | 0.003  (-0.012 – 0.017) | 0.734 | -0.004  (-0.023 – 0.016) | 0.694 | -0.004  (-0.010 – 0.002) | 0.180 | -4.51e-4  (-0.001 – 4.27e-4) | 0.314 | -0.020  (-0.107 – 0.067) | 0.652 |
| Strength | -0.040  (-0.051 – -0.029) | < 0.001 | -0.110  (-0.124 – -0.095) | < 0.001 | -0.036  (-0.040 – -0.031) | < 0.001 | -0.001  (-0.002 – -0.001) | < 0.001 | -0.129  (-0.193 – -0.065) | < 0.001 |
| Time × Strength | -0.007  (-0.015 – 0.001) | 0.073 | -0.005  (-0.015 – 0.005) | 0.335 | 0.001  (-0.002 – 0.004) | 0.547 | 1.34e-4  (-3.19e-4 – 0.001) | 0.562 | 0.006  (-0.039 – 0.051) | 0.795 |
| **Pairwise comparison^a^ (Direct Estimation)** | | | | | | | | | | |
| Unconnected – Low level | 0.027  (-0.003 – 0.058) | 0.065 | 0.020  (-0.013 – 0.053) | 0.354 | -0.001  (-0.011 – 0.008) | 0.896 | -3.24e-4  (-0.002 – 0.002) | 0.989 | -0.010  (-0.197 – 0.177) | 0.978 |
| Unconnected – Medium level | 0.027  (-0.004 – 0.057) | 0.065 | 0.019  (-0.014 – 0.052) | 0.354 | -0.002  (-0.011 – 0.008) | 0.896 | -3.34e-4  (-0.002 – 0.002) | 0.989 | -0.012  (-0.199 – 0.176) | 0.978 |
| Unconnected – High level | 0.024  (-0.007 – 0.054) | 0.083 | 0.017  (-0.016 – 0.050) | 0.354 | -0.003  (-0.012 – 0.007) | 0.896 | -4.43e-4  (-0.002 – 0.001) | 0.989 | -0.019  (-0.206 – 0.168) | 0.978 |
| Low level – Medium level | -0.001  (-0.031 – 0.030) | 0.955 | -0.001  (-0.034 – 0.032) | 0.953 | -4.70e-4  (-0.010 – 0.009) | 0.896 | -9.90e-6  (-0.002 – 0.002) | 0.989 | -0.002  (-0.189 – 0.185) | 0.978 |
| Low level – High level | -0.004  (-0.034 – 0.027) | 0.955 | -0.003  (-0.036 – 0.030) | 0.953 | -0.002  (-0.011 – 0.008) | 0.896 | -1.18e-4  (-0.002 – 0.002) | 0.989 | -0.010  (-0.197 – 0.177) | 0.978 |
| Medium level – High level | -0.003  (-0.034 – 0.028) | 0.955 | -0.003  (-0.035 – 0.030) | 0.953 | -0.001  (-0.011 – 0.008) | 0.896 | -1.09e-4  (-0.002 – 0.002) | 0.989 | -0.008  (-0.195 – 0.179) | 0.978 |

**Table S1.** Results of linear mixed-effects (LME) models assessing longitudinal changes in cortical metrics using the mirrored lesion ROI. The first section reports the interaction between time and connectivity strength. The second section shows pairwise comparisons of cortical change rates across connectivity levels.

^a^: *p*-values in the pairwise comparisons were FDR-corrected.

**10. Diffusion Metrics from Different White Matter Regions and Their Associations with Principal Components of Cortical Degeneration**

|  | PC1 (Direct Estimation) | PC2 (Direct Estimation) | PC1 (Indirect Estimation) | PC2 (Indirect Estimation) |
| --- | --- | --- | --- | --- |
| NAWM FA | 0.348 (0.119 – 0.576)  *p* = 0.004 | 0.002 (-0.243 – 0.248)  *p* = 0.984 | 0.337 (0.105 – 0.568)  *p* = 0.006 | 0.134 (-0.111 – 0.378)  *p* = 0.289 |
| ncNAWM FA | 0.365 (0.138 – 0.592)  *p* = 0.003 | 0.019 (-0.226 – 0.264)  *p* = 0.881 | 0.314 (0.081 – 0.548)  *p* = 0.011 | 0.161 (-0.082 – 0.405)  *p* = 0.199 |
| Tract FA | 0.485 (0.267 – 0.703)  *p* < 0.001 | 0.131 (-0.117 – 0.379)  *p* = 0.303 | 0.362 (0.128 – 0.596)  *p* = 0.004 | 0.095 (-0.155 – 0.345)  *p* = 0.460 |
| NAWM MD | -0.350 (-0.578 – -0.121)  *p* = 0.004 | -0.028 (-0.274 – 0.217)  *p* = 0.821 | -0.247 (-0.486 – -0.008)  *p* = 0.047 | -0.193 (-0.436 – 0.049)  *p* = 0.123 |
| ncNAWM MD | -0.349 (-0.577 – -0.120)  *p* = 0.004 | -0.023 (-0.268 – 0.223)  *p* = 0.857 | -0.245 (-0.484 – -0.006)  *p* = 0.049 | -0.192 (-0.434 – 0.051)  *p* = 0.126 |
| Tract MD | -0.316 (-0.548 – -0.085)  *p* = 0.010 | -0.032 (-0.277 – 0.213)  *p* = 0.800 | -0.247 (-0.485 – -0.008)  *p* = 0.047 | -0.131 (-0.376 – 0.113)  *p* = 0.297 |

**Table S2.** Associations between diffusion MRI metrics derived from different white matter regions (NAWM, ncNAWM, and tract-specific regions) and principal component (PC) scores of cortical degeneration obtained from direct and indirect connectivity estimations. Results are presented as regression coefficients (β) with 95% confidence intervals and *p*-values. NAWM, normal-appearing white matter; ncNAWM, non-connected normal-appearing white matter.

**11. Association between Cortical Degeneration and Clinical Outcomes in RSSI**

|  | **PC1 (Direct)** | | **PC2 (Direct)** | | **PC1 (Indirect)** | | **PC2 (Indirect)** | |
| --- | --- | --- | --- | --- | --- | --- | --- | --- |
|  | β (95% CI) | *p*-value | β (95% CI) | *p*-value | β (95% CI) | *p*-value | β (95% CI) | *p*-value |
| Δ mRS | 0.22  (-0.22 – 0.66) | 0.337 | 0.09  (-0.20 – 0.39) | 0.539 | 0.15  (-0.05 – 0.35) | 0.148 | -0.28  (-0.57 – 0.01) | 0.066 |
| Δ MoCA | -0.75  (-1.97 – 0.47) | 0.235 | 0.05  (-0.77 – 0.88) | 0.898 | 0.03  (-0.53 – 0.59) | 0.916 | 0.64  (-0.19 – 1.47) | 0.136 |
| Δ STT-A | 2.15  (-9.89 – 14.19) | 0.728 | 12.94  (5.50 – 20.38) | 0.001 | 1.24  (-4.25 – 6.74) | 0.659 | 9.47  (1.56 – 17.39) | 0.022 |
| Δ STT-B | 11.02  (-7.56 – 29.60) | 0.249 | 0.001  (-12.54 – 12.54) | 1.000 | -5.32  (-13.79 – 3.16) | 0.223 | 14.28  (1.92 – 26.63) | 0.027 |
| Δ HAMA | -2.38  (-4.30 – -0.47) | 0.017 | 0.22  (-1.11 – 1.55) | 0.744 | -0.74  (-1.64 – 0.15) | 0.108 | -0.95  (-2.30 – 0.39) | 0.169 |
| Δ HAMD | -0.85  (-2.66 – 0.97) | 0.365 | 0.58  (-0.64 – 1.79) | 0.355 | 0.28  (-0.55 – 1.11) | 0.510 | -0.70  (-1.93 – 0.54) | 0.273 |

**Table S2.** Results of general linear models (GLM) assessing the association between cortical degeneration and changes in clinical outcomes in RSSI patients. Principal component scores were used as independent variables, and changes in clinical measures (follow-up minus baseline) were used as dependent variables. Models predicting ΔmRS were adjusted for age, sex, vascular risk factor (VRF) score, and follow-up interval. All other models additionally adjusted for years of education.

mRS, Modified Rankin Scale; MoCA, Montreal Cognitive Assessment-Beijing version; STT, Shape Trail Test; HAMA, Hamilton Anxiety Rating Scale; HAMD, Hamilton Depression Rating Scale.

**Reference**

Duering, M., Righart, R., Csanadi, E., Jouvent, E., Hervé, D., Chabriat, H., Dichgans, M., 2012. Incident subcortical infarcts induce focal thinning in connected cortical regions. Neurology 79, 2025-2028.

Duering, M., Righart, R., Wollenweber, F.A., Zietemann, V., Gesierich, B., Dichgans, M., 2015. Acute infarcts cause focal thinning in remote cortex via degeneration of connecting fiber tracts. Neurology 84, 1685-1692.

Jones, D.K., 2008. Tractography gone wild: probabilistic fibre tracking using the wild bootstrap with diffusion tensor MRI. IEEE Trans Med Imaging 27, 1268-1274.

Kuceyeski, A., Kamel, H., Navi, B.B., Raj, A., Iadecola, C., 2014. Predicting future brain tissue loss from white matter connectivity disruption in ischemic stroke. Stroke 45, 717-722.

Li, H., Jacob, M.A., Cai, M., Duering, M., Chamberland, M., Norris, D.G., Kessels, R.P.C., de Leeuw, F.E., Marques, J.P., Tuladhar, A.M., 2023. Regional cortical thinning, demyelination and iron loss in cerebral small vessel disease. Brain 146, 4659-4673.

Lin, M., Wang, S., Hong, H., Zhang, Y., Xie, L., Cui, L., Liu, L., Jiaerken, Y., Yu, X., Zhang, M., De Luca, A., Zhang, R., Huang, P., 2025. Longitudinal changes in white matter free water in cerebral small vessel disease: Relationship to cerebral blood flow and white matter fiber alterations. J Cereb Blood Flow Metab 45, 932-944.

Smith, R.E., Tournier, J.D., Calamante, F., Connelly, A., 2013. SIFT: Spherical-deconvolution informed filtering of tractograms. Neuroimage 67, 298-312.

Wang, Y., Ye, C., Pan, R., Tang, B., Li, C., Liu, J., Tao, W., Zhang, X., Yang, T., Yan, Y., Jiang, S., Lui, S., Wu, B., 2025. Cognitive implications and associated transcriptomic signatures of distinct regional iron depositions in cerebral small vessel disease. Alzheimers Dement 21, e70196.

Zanon Zotin, M.C., Yilmaz, P., Sveikata, L., Schoemaker, D., van Veluw, S.J., Etherton, M.R., Charidimou, A., Greenberg, S.M., Duering, M., Viswanathan, A., 2023. Peak Width of Skeletonized Mean Diffusivity: A Neuroimaging Marker for White Matter Injury. Radiology 306, e212780.
